# Cervical microbiome is altered in cervical intraepithelial neoplasia after loop electrosurgical excision procedure in china

**DOI:** 10.1038/s41598-018-23389-0

**Published:** 2018-03-21

**Authors:** Hongwei Zhang, Jiaqi Lu, Yingying Lu, Qingqing Cai, Haiou Liu, Congjian Xu

**Affiliations:** 1 Obstetrics and Gynecology Hospital, Fudan University, Shanghai, China; 2 Department of Obstetrics and Gynecology of Shanghai Medical School, Fudan University, Shanghai, China; 3 Shanghai Key Laboratory of Female Reproductive Endocrine Related Diseases, Shanghai, China

## Abstract

Although human papillomavirus (HPV) infection is a major cause leading to the development of cervical intraepithelial neoplasia (CIN), the relationship between genital microbiome and HPV persistence/clearance is not well established. Loop electrosurgical excision procedure (LEEP) is one of standard treatments of CIN 2/3 globally, yet little is known about how the LEEP influence genital microbiota. We conducted a prospective study of 26 patients with CIN2/3 who underwent analysis of cervical microbiome before and after 3 months of LEEP treatment. Cervical swabs were collected, and microbiomes were analyzed by 16S ribosomal RNA gene sequencing. A decrease of cervical microbial diversity was observed after 3 months of LEEP treatment. Notably, a significant shift from community type of a *Prevotella*-containing and lack of a consistent dominant species to *lactobacillus iners* dominated microbiome correlated with LEEP. Particularly, *Leptotrichia* and *clostridium* were further decreased after LEEP treatment (*P* = 0.049 and *P* = 0.002, respectively). Our results suggest that the cervical microbiome is altered after LEEP treatment in patients with CIN2/3. Further studies with larger sample sizes are needed to validate these findings.

## Introduction

Cervical intraepithelial neoplasia (CIN), a premalignant lesion of the uterine cervix, can be histologically divided into three stages (1, 2, and 3)^1^. Persistent infection with high-risk human papillomavirus (HPV) is the main event leading to the development of CIN and cervical cancer^2^. However, the factors promoting HPV persistence and triggering carcinogenetic pathways remain elusive. It has been recently proposed that local microbial communities may affect the acquisition and persistence of HPV, and subsequent development of cervical cancer^3^.

The degree of microbiome complexity influences the pathogenicity of HPV infection in the female genital tract. A condition characterized by a decrease in *Lactobacillus* with a concomitant increase in anaerobic bacteria (e.g., *Gardnerella*, *Prevotella*, and *Clostridiales*) is associated with increased risk of delayed clearance of HPV infection^4^. Consistently, a cervical microbiome determined by paucity of *L. crispatus* with concomitant occupied by *A. vaginae, G. vaginalis* and *L. iners* was associated with CIN risk^5,6^. Another report suggested that the predominant cervical bacterial in the woman with squamous intraepithelial lesions (SIL) was *Leptotrichia amnionii*^7^. Studies have demonstrated that microbial dysbiosis may be a risk factor for the development of HPV infection and cervical neoplasia^5,8,9^.

Women with CIN2/3 are at high risk of developing invasive cervical cancer, whereas the risk is very low in women with conservative treatment^10^. A loop electrosurgical excision procedure (LEEP), to remove the dysplastic cells and allow new cells to replace the old ones, is an effective treatment to reduce the risk of cervical cancer. It is now widely recognized on the clearance of HPV infection after the successful treatment of CIN using LEEP^11^. These results raise intriguing questions about cervical microbiome response underlying the LEEP.

The purpose of the current study was to explore the extent of changes in the cervical microbiome following LEEP treatment. To reveal the effect of LEEP intervention on cervical microbiota in patients with CIN2/3, we compared the microbial communities before and after 3 month of LEEP therapy. The results indicated that LEEP treatment altered the cervical microbiome in patients with CIN2/3.

## Results

### LEEP’s impact on the cervical microbiome

**Figure 1:**
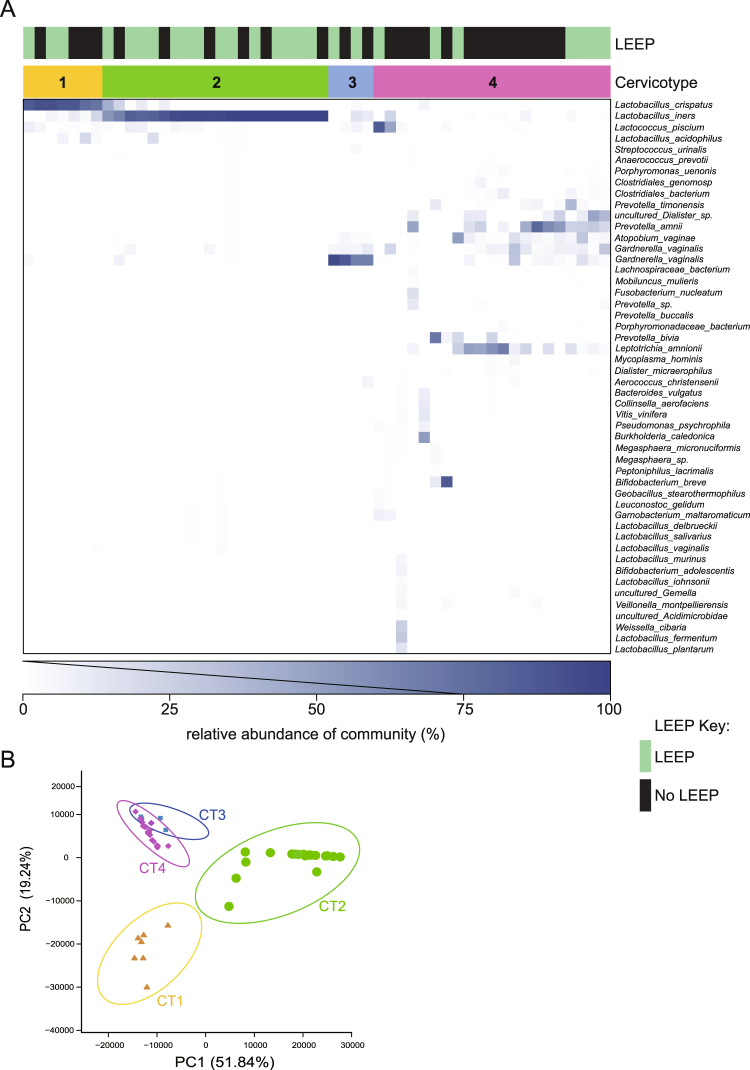
16S rRNA sequence analysis of cervical swabs reveals four distinct bacterial community structures. (**A**) Heatmap of bacterial taxa identified by 16S rRAN V3-4 sequencing of cervical swabs collected from 26 patients with or without LEEP procedure. Cervicotypes (CTs) were determined based on the dominant species: non-*iners Lactobacillus* (i.e., higher percentage of *Lactobacillus crispatus*) (CT1), *Lactobacillus iners* (CT2), *Gardnerella* (CT3), and mixed microbial community containing *Prevotella* (CT4). (**B**) Principal component analysis (PCA) plot constructed from 52 samples. The first two principal components (PC1 and PC2) can explain 71.08% of the data variance. Different colors denote 4 distinct Cts. P = 0.001 for CTs.

In total 1,914,331 reads were obtained from 52 samples with an average number of reads per sample of 36,814 and the mean and medium read lengths of 445 and 453 bp respectively. Using bacterial genera sequence data, samples were classified according to their bacterial communities consistent with previously described cervical microbiome as cervicotypes (CTs)^12^; CT1: primarily composed of non-*iners Lactobacillus* (i.e., higher percentage of sequencing reads from non-*iners Lactobacillus* than *L. iners*, *Gardnerella*, or *Prevotella*), CT2: *Lactobacillus iners* dominated, CT3: *Gardnerella* dominated, CT4: lack of a consistent dominant species but communities all included *Prevotella* (Fig. 1A). To further explore the relationship between 4 distinct CTs, a PCA analysis was performed using the species-level taxonomic profiles. As shown in Fig. 1B and Supplementary Fig. S1, the first two principal components, representing 71.08% of the variance, classified the 52 samples into three groups, though CT3 and CT4 are a continuum. The analysis of similarity (ANOSIM) test using Bray-Curtis dissimilarity showed that the observed cluster patterns were significant (R^2^ = 0.5676, *P* = 0.001). Although the clusters were not clearly separated by LEEP surgery, most women with LEEP surgery carried *L. iners* as the dominant members.

The rates and frequency of the different CTs were compared with and without LEEP surgery (Table 1). We found that 53.9% patients without consistent predominant bacterial taxon, though each community was found to have *prevotella* (CT4) before LEEP. Reduced rate of CT4 from 53.9% to 26.9% were observed in patients with LEEP surgery (*P* = 0.048). Conversely, frequency of CT2 in patients with LEEP increased from 23.1 to 53.9% (*P = *0.023). These analyses are suggestive of association between *Prevotella* and cervical disease^13^.

**Table 1:** Rates of each CT according to LEEP.

Characteristics	CT1 *L. crispatus* n/N (%)	CT2 *L. iners* n/N (%)	CT3 *Gardnerella* n/N (%)	CT4 Diverse species n/N (%)	
NO LEEP	4/26 (15.4)	6/26 (23.1)	2/26 (7.7)	14/26 (53.9)	
LEEP	3/26 (11.5)	14/26 (53.9)	2/26 (7.7)	7/26 (26.9)	
*P value*	1.000	0.023	1.000	0.048	0.128

CT: cervicotypes; CT1: primarily composed of non-*iners Lactobacillus* (i.e., higher percentage of *Lactobacillus crispatus*); CT2: *Lactobacillus iners* dominated; CT3: *Gardnerella* dominated; CT4: diversity species, but communities all included *Prevotella*; *P* value calculated using Fishers exact test.

**Figure 2:**
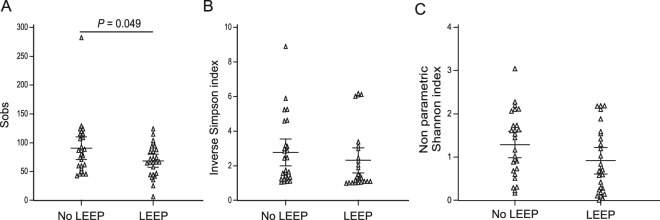
Cervical microbiome richness and diversity indices associated with LEEP status. The number of species observed decreased in patients with LEEP (**A**). Diversity, as assessed by Inverse Simpson (**B**) and non-parametric Shannon (**C**) indices followed the same pattern. Kruskall-Wallis test (Dunn’s post hoc).

Species richness (Fig. 2A) were higher in patients without LEEP compared to those with LEEP (*P* = 0.049). Microbiota diversity was also found to be higher in patients without LEEP in compared with those with LEEP but this was not statistically significant (Fig. 2B,C). Consistent with increased rates of CT4 in patients without LEEP, species alpha-diversity (Supplementary Fig. S2B,C) were higher in CT4, compared to other CTs particularly CT1 (*P* < 0.001) and CT3 (*P* = 0.035 and *P = *0.005), while there was no different in species richness between CTs (Supplementary Fig. S2A).

**Figure 3:**
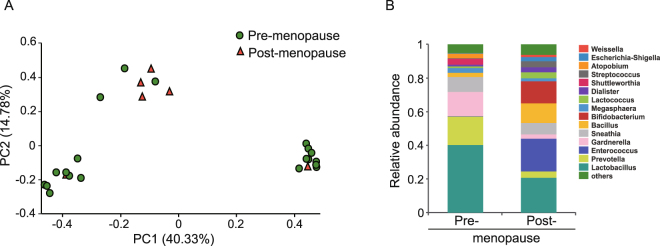
Principal coordinates analysis (PCoA) and genus level relative abundance for 26 samples from the patients before LEEP. (**A**) Bray-Curtis dissimilarity PCoA was used to generate ordination of beta-diversity in two dimensions. Principal coordinates 1 and 2 (PC1 and PC2) explain 40.33% and 14.78% of the variance in Bray-Curtis dissimilarity respectively (x and y axes). Samples are colored according to the menopause status. (**B**) Relative abundance was shown for the top fifteen genus.

Furthermore, to investigate whether the pre- and post-menopausal status altered the microbial communities across the LEEP treatments, we performed beta diversity analysis by using Principal Coordinates Analysis (PCoA) based on Bray-Curtis dissimilarities. Using Bray-Curtis dissimilarity significant differences were observed between pre- and post-menopausal groups before LEEP treatment (ANOSIM, R^2^ = 0.224, *P* = 0.023) (Fig. 3A). In pre-menopausal group before LEEP, four major taxa at genus level were observed, *Lactobacillus* (40.2%), *Prevotella* (17%), *Gardnerella* (14.6%), and *Sneathia* (8.7%) (Fig. 3B). In post-menopausal group before LEEP, a similar composition of major cervical bacteria was observed, but with altered proportions, involving the replacement of *Lactobacillus* (20.7%), *Enterococcus* (19.4%), *Bacillus* (11.7%), and *Sneathia* (6.7%). In contrast, microbial diversity was not observed between pre and postmenopausal groups after treated with LEEP (Supplementary Fig. S3A). After receiving LEEP therapy, premenopausal women exhibited increase in *Lactobacillus* up to 66.8%. Similar to the effect of LEEP on postmenopausal women, increase of *Lactobacillus* up to 35% was observed (Supplementary Fig. S3B).

### Identification of cervical microbiota composition markers correlated with LEEP

**Figure 4:**
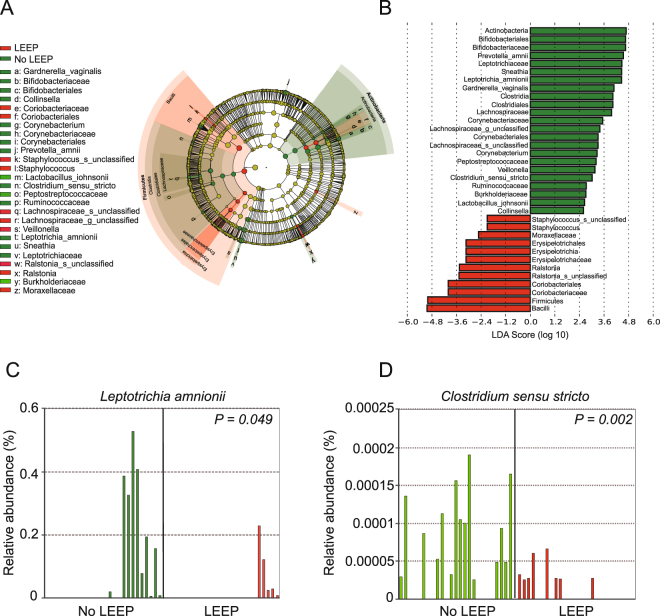
Bacterial taxonomic groups discriminate between before and following LEEP procedure. (**A**) Differentially abundant microbial clades and nodes according to LEEP surgery were identified using LEfSe analysis and presented as a cladogram. (**B**) Histogram of the LDA scores was used to features differentially abundant between LEEP and no LEEP state. The cervical microbiome of patients following LEEP surgery was enriched with *Bacilli and Firmicutes*, whereas those before LEEP were comparatively enriched *Actinobacteria* and *Bifidobacteriales*. Relative abundance bar charts for individual samples highlight decreased numbers of *Leptotrichia amnion* (**C**) and *Clostridium sensu strict* (**D**) after LEEP (Welchi’s t-test).

To identify bacteria specifically associated with LEEP status, we performed linear discriminant analysis (LDA) with effect size (LEfSe) modeling. Bacterial families, including *Bifidobacteriaceae, Lachnospiraceae, Leptotrichiaceae*, and *Peptostreptococcaceae* were enriched in No LEEP samples. Bacterial families, including *Erysipelotrichaceae* and *Coriobacteriaceae* were enriched in LEEP samples. Furthermore, samples from No LEEP were enriched in *Sneathia, Collinsella, Veillonella, Clostridia*, and unclassified genus belonging to *Lachnospiraceae* (effect size >2.0; *P* < 0.05). (Fig. 4A,B). Following LEEP surgery was characterized by decreased levels of *Leptotrichia amnionii* (*P* = 0.049), and *Clostridium sensu stricto* (*P* = 0.002) (Fig. 4C,D).

## Discussion

In the current study, we applied a 16S rRNA sequencing approach to a longitudinal group of patients analyzed before and after LEEP therapy. An alteration of cervical microbiome was observed in patients of CIN2/3 follow-up three months of LEEP therapy. The cervical microbiome after LEEP reflected reduced species richness as well as a significant shift from community type of a *Prevotella*-containing and lack of a consistent dominant species to *L. iners*-dominated microbiome.

It is well established that healthy vaginal microbial communities are dominated with low bacterial diversity, while vaginal dysbiosis is often characterized by high diversity bacterial populations with increased anaerobic bacteria^14^. The increased diversity of vaginal microbiota has been associated with bacterial vaginosis^15^, preterm birth^16^, acquisition of sexually transmitted infections^14^, and persistence and development of cervical cancer^6,17^. There are also several reports regarding the association between cervical microbiomes and genital diseases. A significant difference in microbial diversity was found between non-cervical lesions with HPV negative women and those with squamous intraepithelial lesions and cervical cancer in a recent study^7^. The cervical mucosal community dominated by *L. iners* and unclassified *Lactobacillus spp*. was associated with higher grade cervical intraepithelial neoplasia in women infected with HR HPV^4^. As LEEP is one of effective therapies to remove of cervical dysplasia, we observed that the cervical community type dominated by *L. iners* in patients with CIN2/3 after LEEP.

Ravel *et al*.^18^ demonstrated that vaginal microbiota can be categorized into five community state types; four are dominated by different species of *Lactobacillus* species (*L. iners, L. crispatus, L. gasseri, L. jensenii*), but one is devoid of *Lactobacillus spp*. and instead enriched with strict anaerobic species including *Gardnerella, Megasphera, Sneathia and Prevotella*, which have subsequently been used by numerous other studies^6,19,20^. However, the cervical microbiota profiles have been categorized to four distinct cervicotypes^12^. Consistently, we identified CT4 (mixed microbial community containing *Prevotella*) prominent in patients with CIN2/3 before LEEP, whereas CT2 (dominated by *Lactobacillus iners*) was enriched in patients after LEEP.

It has been proved that the bacterial community within the female genital tract has a profound impact in human’s health, since the cervical microbiome is involved in the modulation of the inflammatory immune response^12,14^. The predominant cervical microbiome in women with normal cytology was *Lactobacillus crispatus* and *Lactobacillus iners*, whereas with squamous intraepithelial lesions was *Sneathia spp*^7^. *Lactobacillus crispatus*-rich cervical microbiota were found to manifest the lowest inflammation, while *Prevotella*-containing and to a lesser extent *Gardnerella*-dominant microbiota, correlated with multiple pro-inflammatory mucosal cytokines even in the absence of overt sexually transmitted diseases^12^. *Prevotella* is a key vaginal species has negative impacts on women’s health^13^.

*Lactobacillus crispatus* is associated with a relatively stable state in the cervico-vaginal environment, whereas *L. iners* is associated with increased risk of transiting from normal to bacterial vaginosis (BV)^3,21,22^. On the other hand, *L. iners* has been reported to become a predominant part of the microbial community when the vaginal microbiota transitions between abnormal and normal states^23^. In our study, *L. iners* was the most abundant species in the cervix of women with LEEP. Thus, LEEP is not sufficient to restore a healthy cervical bacterial community. Recent advances in the knowledge of human microbiome have implications for the potential use of probiotics and prebiotics to restore human healthy microbial communities in promoting genital health in women^24,25^.

*Sneathia spp. (L. amnionii)* was found to be correlated with HPV infection in a Korean twin cohort^26^. *Sneathia amnii* has been identified as a possible microbiological marker associated cervical cancer in HPV-positive subjects^27^. In our study, we found that *Sneathia amnii* was enriched in patients with CIN2/3 before LEEP.

Although our results revealed the alterations of cervical microbiome after LEEP in patients with CIN2/3, there are several limitations needed to be addressed in the future studies. First, we were not able to observe microbiome variations among patients with different outcome after LEEP due to its small number of patients and relative short follow up period. Second, the impact of mucosal microbiota alteration on the inflammation cytokine profiles should be observed.

## Materials and Methods

### Patients

We prospectively recruited 50 patients who underwent LEEP for CIN2/3 at our department of Cervical Disease of Obstetrics and Gynecology hospital of Fudan University from June 2016 to Jan 2017. Inclusion criteria were included, not pregnant, no previous history of cervical or other lower genital cancer, no previous hysterectomy, or destructive therapy of the cervix, had no current use of hormonal or barrier contraceptive products, vaginal douching, tobacco or alcohol abuse, not HIV or hepatitis B/C positive or autoimmune disorders or antibiotics/probiotics (oral or topical) within six months prior to sample collection, and had no sexual activity in the previous days of the sampling. Cervical mucus samples were collected just before and after three months of LEEP. Of the 50 patients, 2 (4%) underwent a hysterectomy during the study period, primarily due to invasive cancer, and 22 (44%) patients had no follow-up visit after LEEP. Finally, 26 patients enrolled in this study. All of the subjects were Chinese ethnicity whose age ranged between 25–68 years (including six post-menopausal women), and detailed patient characteristics are listed in Table 2 and Supplementary Table S1. Epidemiological data, pathological reports, high-risk (HR)-HPV test results, and follow-up data from the medical archives were reviewed. Patients were positive for HR-HPV (any one of 13 types of HR-HPV, HPV 16, 18, 31, 33, 35, 39, 45, 51, 52, 56, 58, 59, and 68) based on the Hybrid Capture 2 assay (HC2; Qiagen, Gaithersburg, Maryland). Written informed consent was obtained from each participant following protocols approved by the Ethics Committee of the Affiliated Obstetrics and Gynecology Hospital of Fudan University. All experiments were performed in accordance with the approved guidelines.

**Table 2:** Patients’ characteristics.

Characteristic	Values
Age (yr)	39 (25–68)
**Pap test**	
LSIL	3 (11.5%)
HSIL	23 (88.5%)
**HPV type (preoperative)**	
Negative	1 (3.8%)
Type 16	14 (53.8%)
Type 18	3 (11.6%)
Other high-risk	8 (30.8%)
**Surgical results**	
Complete excision of the lesion	24 (92.3%)
No entirely excised lesion	2 (7.7%)
**Pathology**	
CIN2	1 (3.8%)
CIN3	25 (96.2%)
**HPV testing 3 months after LEEP**	
Positive	1 (3.8%)
Negative	25 (96.2%)

Values are presented as median (range) or number (%).

CIN, cervical intraepithelial neoplasia; HPV, human papillomavirus; HSIL, high-grade squamous intraepithelial lesion; LEEP, loop electrosurgical excision procedure; LSIL, low-grade squamous intraepithelial lesion.

### Sample collection and DNA extraction

Cervical mucus samples were collected using swabs and stored at −80 °C within 3 hours after sampling. Microbial DNA was extracted using a QIAamp Fast DNA Stool Mini Kit (Qiagen Inc., Hilden, Germany) according the manufacturer’s instructions. The concentration of bacterial DNA was measured using Nanodrop 2000 (Thermo Scientific).

### 16S ribosomal RNA sequencing

The V3-V4 hypervariable regions of the bacteria’s 16S ribosomal RNA (rRNA) gene were amplified by PCR with barcode-indexed primers 338 F (5′- ACTCCTACGGGAGGCAGCAG-3′) and 806 R (5′-GGACTACHVGGGTWTCTAAT-3′) using FastPfu polymerase. Purified amplicons were pooled in equimolar concentrations, and paired-end sequenced using an Illumina MiSeq (Illumina, San Diego, California). The raw reads were deposited into the NCBI Sequence Read Archive (SRA) database (Accession Number: SRP114960).

### Microbial analysis

Sequencing reads were demultiplexed and filtered by Trimmomatic and merged by FLASH with the following criteria: (1) The reads were truncated at any site receiving an average quality score <20 over a 50 bp sliding window. (2) Primers were exactly matched allowing 2 nucleotides mismatching, and reads containing ambiguous bases were removed. (3) Sequences whose overlap longer than 10 bp were merged according to their overlap sequence. Paired-end reads were overlapped using PANDAseq with a required overlap length of >300 bp^28^. Reads less than 100 nucleotides or lacking a correct primer were removed^29^. The 16S rRNA sequencing data were processed using the Quantitative Insights Into Microbial Ecology platform (QIIME, V.1.9.1)^30^. Operational taxonomic units (OTUs) were clustered with 97% similarity cutoff using UPARSE (version 7.1 http://drive5.com/uparse/) and chimeric sequences were identified and removed using UCHIME^31^. The taxonomy of each 16S rRNA gene sequence was analyzed by RDP Classifier algorithm (http://rdp.cme.msu.edu/) against the Silva (SSU123) 16S rRNA database using confidence threshold of 70%. A total of 8,600,000 sequences clustered to 652 OTUs were obtained after quality filtering. Alpha diversity was analyzed with mother^32^. Richness of each sample was calculated with the Sobs index and diversity accounting for both relative abundance and evenness was evaluated with Invsimpson and Shannon index. The Principal Component Analysis (PCA) was performed by the R package ade4. Each coordinate on the score plot represents an individual sample. Principal Coordinates Analysis (PCoA) based on Bray-Curtis dissimilarities were performed. Permutational Multivariate Analysis of Variance Using Distance Matrices (PERMANOVA) and Analysis of Similarities (ANOSIM) were carried out using the ‘adonis’ and ‘anosim’ functions in the ‘vegan’ package, respectively, with Bray-Curtis dissimilarities and 999 permutations.

### Statistical Analysis

Examination of statistical differences between cervical microbiota was performed at bacterial genera and species levels using the Statistical Analysis of Metagenomic Profiles software package. Ward’s linkage hierarchical clustering analysis (HCA) of bacterial genera was performed using a clustering density threshold of 0.75. Bacterial species data were classified into CTs as described by Anahtar *et al*.^12^: CT I (non-*iners lactobacillus; high percentage of Lactobacillus crispatus*), CT II (*L. iners*), CT III (*Gardnerella*), and CT IV (mixed bacterial species containing *Prevotella*). The effects of LEEP on bacterial genera, number of species observed, and α diversity were assessed using one-way ANOVA, Kruskal-Wallis test, and Dunn’s multiple comparison test, where appropriate. The LEfSe method^33^ characterized differentially abundant taxonomic features before and 3 months after LEEP. An α value of 0.05 was used for factorial Kruskal-Wallis test between classes, and a threshold of 2.0 was used for logarithmic LDA score for discriminative features. Fisher’s exact test was used to comparing categorical data among two or more groups. *P* values are two-sided. The analyses were performed with R packages (V.2.15.3) and Prism (GraphPad).

## Electronic supplementary material

Supplementary Information
