# Supplementary material for: Cervical microbiome is altered in cervical intraepithelial neoplasia after loop electrosurgical excision procedure in china
Source: Sci Rep. 2018 Mar 21;8:4923. doi: 10.1038/s41598-018-23389-0 (PMC5862885; doi:10.1038/s41598-018-23389-0)
Supplement: Supplementary file 1 — Supplementary Information [file 41598_2018_23389_MOESM1_ESM.docx]

**Supplementary Information**

**Cervical microbiome is altered in cervical intraepithelial neoplasia after loop electrosurgical excision procedure in china**

Hongwei Zhang^1^^,¶^, Jiaqi Lu^1,¶^, Yingying Lu^1^, Qingqing Cai^3^, Haiou Liu^3*^, Congjian Xu^1, 2,3*^

Correspondence and requests for materials should be addressed to H. Liu ([liuhaiou@fudan.edu.cn](mailto:liuhaiou@fudan.edu.cn)) or C. Xu (xucongjian@fudan.edu.cn)

**Supplementary Table S1. Characteristics of enrolled patients.**

| ID  Patient | Age | Menopause status | HPV status pretreatment | HPV types at pretreatment | PAP test at Pretreatment | Colposcopy at Pretreatment | CT at Pretreatment | HPV status at 3-month follow up | CT at follow-up |
| --- | --- | --- | --- | --- | --- | --- | --- | --- | --- |
| 1 | 36 | Pre- | Positive | 18 | HSIL | CIN3 | 1 | Negative | 1 |
| 2 | 35 | Pre- | Positive | 16 | HSIL | CIN3 | 1 | Negative | 1 |
| 3 | 43 | Pre- | Positive | 16 | LSIL | CIN3 | 1 | Negative | 2 |
| 4 | 34 | Pre | Positive | 16 | HSIL | CIN3 | 1 | Negative | 2 |
| 5 | 39 | Pre- | Positive | 16 | HSIL | CIN3 | 2 | Negative | 2 |
| 6 | 26 | Pre- | Positive | 16 | HSIL | CIN3 | 4 | Negative | 2 |
| 7 | 45 | Pre- | Positive | 16 | HSIL | CIN3 | 4 | Negative | 2 |
| 8 | 39 | Pre- | Positive | 18 | HSIL | CIN3 | 3 | Negative | 2 |
| 9 | 40 | Pre- | Positive | 16 | HSIL | CIN3 | 2 | Negative | 2 |
| 10 | 53 | Post- | Positive | 52 | HSIL | CIN3 | 2 | Positive | 2 |
| 11 | 26 | Pre- | Positive | 52 | HSIL | CIN3 | 3 | Negative | 2 |
| 12 | 45 | Pre- | Negative | **-** | HSIL | CIN3 | 2 | Negative | 2 |
| 13 | 25 | Pre- | Positive | 16 | HSIL | CIN3 | 4 | Negative | 2 |
| 14 | 43 | Pre- | Positive | 16 | HSIL | CIN3 | 2 | Negative | 4 |
| 15 | 36 | Pre- | Positive | 16 | HSIL | CIN3 | 4 | Negative | 3 |
| 16 | 58 | Post- | Positive | 18 | HSIL | CIN3 | 4 | Negative | 4 |
| 17 | 60 | Post- | Positive | 58 | LSIL | CIN3 | 4 | Negative | 4 |
| 18 | 53 | Post- | Positive | 58 | HSIL | CIN3 | 4 | Negative | 4 |
| 19 | 34 | Pre- | Positive | 16 | HSIL | CIN3 | 4 | Negative | 4 |
| 20 | 32 | Pre- | Positive | 16 | HSIL | CIN3 | 4 | Negative | 4 |
| 21 | 48 | Post- | Positive | 31,52,58 | HSIL | CIN3 | 4 | Negative | 4 |
| 22 | 36 | Pre- | Positive | 58 | HSIL | CIN3 | 4 | Negative | 1 |
| 23 | 29 | Pre- | Positive | 52 | HSIL | CIN3 | 4 | Negative | 2 |
| 24 | 48 | Pre- | Positive | 58 | HSIL | CIN3 | 4 | Negative | 3 |
| 25 | 68 | Post- | Positive | 16 | HSIL | CIN3 | 4 | Negative | 2 |
| 26 | 38 | Pre | Positive | 16 | LSIL | CIN2 | 2 | Negative | 2 |


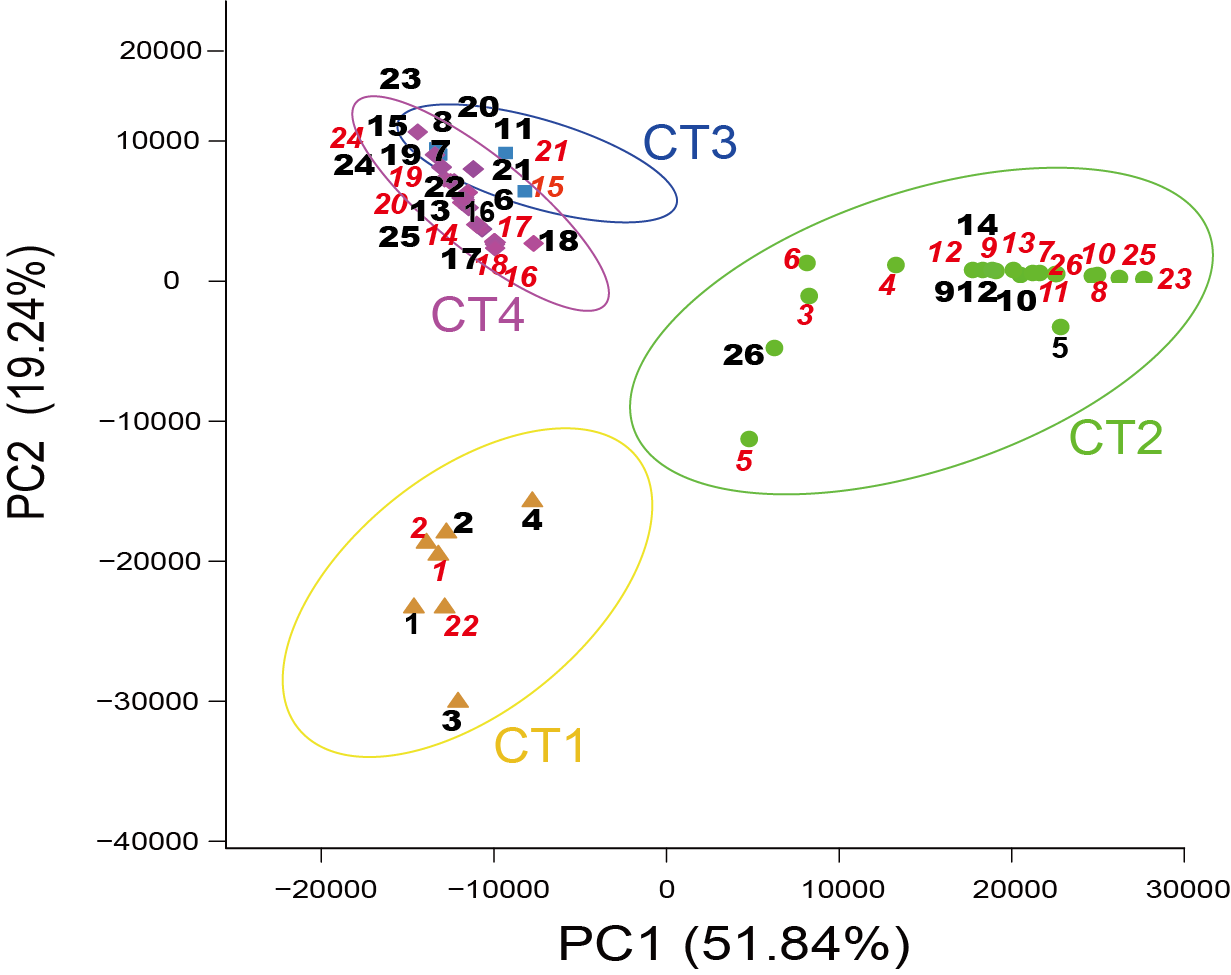


**Supplementary Figure S1. Principal component analysis (PCA) plot constructed from 52 samples.** The first two principal components (PC1 and PC2) can explain 71.08% of the data variance. Different colors denote 4 distinct Cts. P=0.001 for CTs, sample ID marked before LEEP (black bold number) and after LEEP (red italic number).


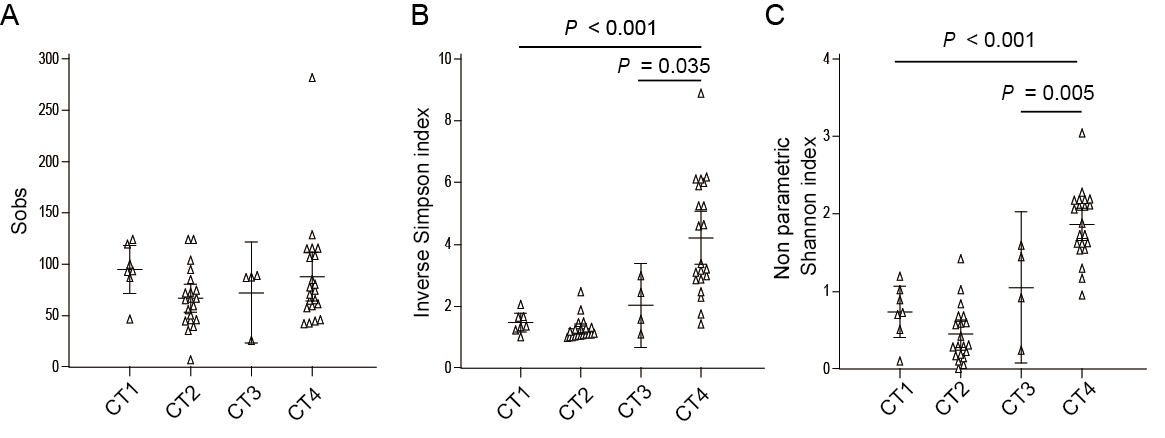


**Supplementary Figure S2.** **Analysis of richness and diversity indices with attributed CTs for the patient cohort.** A similar number of species observed in samples classified as different CTs (A). Diversity was significantly higher in CT4 classified samples as assessed by the Inverse Simpson (B) and non-parametric Shannon (C) indices compared to CT1 (P < 0.001) and CT3 (*P* = 0.035 and *P* = 0.005). Kruskall-Wallis test (Dunn’s post hoc).


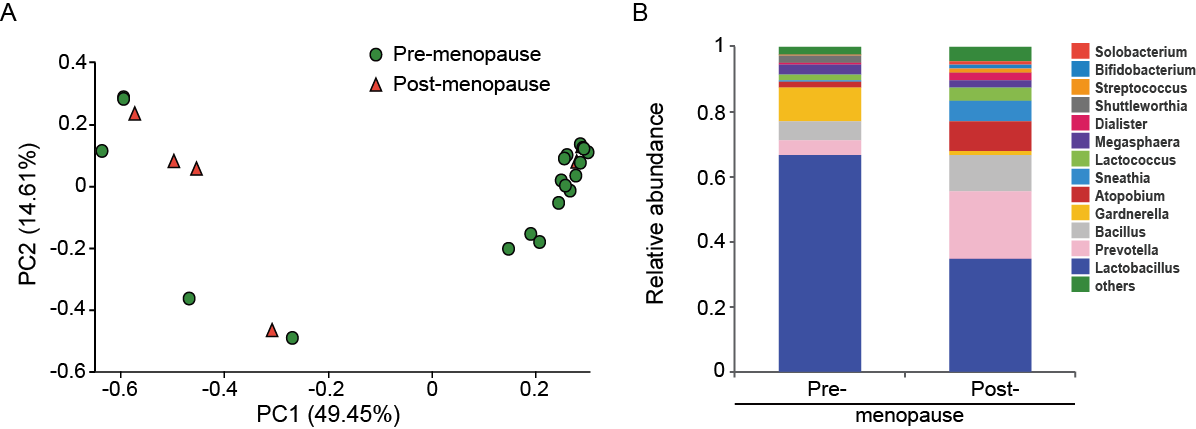


**Supplementary Figure S3. Principal coordinates analysis (PCoA) and genus level relative abundance for 26 samples from the patients after LEEP.** (A) Bray-Curtis dissimilarity PCoA was used to generate ordination of beta-diversity in two dimensions. Principal coordinates 1 and 2 (PC1 and PC2) explain 49.45% and 14.61% of the variance in Bray-Curtis dissimilarity respectively (x and y axes). Samples are colored according to the menopause status. (B) Relative abundance was shown for the top fifteen genus.
